# Expression of Regulatory Platelet MicroRNAs in Patients with Sickle Cell Disease

**DOI:** 10.1371/journal.pone.0060932

**Published:** 2013-04-12

**Authors:** Shilpa Jain, Maria G. Kapetanaki, Nalini Raghavachari, Kimberly Woodhouse, Guoying Yu, Suchitra Barge, Claudia Coronnello, Panayiotis V. Benos, Gregory J. Kato, Naftali Kaminski, Mark T. Gladwin

**Affiliations:** 1 Division of Pediatric Hematology-Oncology, Children’s Hospital of Pittsburgh, University of Pittsburgh Medical Center, Pittsburgh, Pennsylvania, United States of America; 2 Vascular Medicine Institute, University of Pittsburgh, Pittsburgh, Pennsylvania, United States of America; 3 Dorothy P. and Richard P. Simmons Center for Interstitial Lung Disease, Division of Pulmonary, Allergy and Critical Care Medicine, University of Pittsburgh School of Medicine, Pittsburgh, Pennsylvania, United States of America; 4 Genomics Core Facility, National Heart, Lung, and Blood Institute, National Institutes of Health, Bethesda, Maryland, United States of America; 5 Department of Computational and Systems Biology, University of Pittsburgh School of Medicine, Pittsburgh, Pennsylvania, United States of America; 6 Fondazione Ri.Med, Palermo, Italy; 7 Sickle Cell Vascular Disease Section, Pulmonary and Vascular Medicine Branch, National Heart, Lung, and Blood Institute, National Institutes of Health, Bethesda, Maryland, United States of America; 8 Division of Pulmonary, Allergy and Critical Care Medicine, University of Pittsburgh School of Medicine, Pittsburgh, Pennsylvania, United States of America; Duke University, United States of America

## Abstract

**Background:**

Increased platelet activation in sickle cell disease (SCD) contributes to a state of hypercoagulability and confers a risk of thromboembolic complications. The role for post-transcriptional regulation of the platelet transcriptome by microRNAs (miRNAs) in SCD has not been previously explored. This is the first study to determine whether platelets from SCD exhibit an altered miRNA expression profile.

**Methods and Findings:**

We analyzed the expression of miRNAs isolated from platelets from a primary cohort (SCD = 19, controls = 10) and a validation cohort (SCD = 7, controls = 7) by hybridizing to the Agilent miRNA microarrays. A dramatic difference in miRNA expression profiles between patients and controls was noted in both cohorts separately. A total of 40 differentially expressed platelet miRNAs were identified as common in both cohorts (p-value 0.05, fold change>2) with 24 miRNAs downregulated. Interestingly, 14 of the 24 downregulated miRNAs were members of three families - miR-329, miR-376 and miR-154 - which localized to the epigenetically regulated, maternally imprinted chromosome 14q32 region. We validated the downregulated miRNAs, miR-376a and miR-409-3p, and an upregulated miR-1225-3p using qRT-PCR. Over-expression of the miR-1225-3p in the Meg01 cells was followed by mRNA expression profiling to identify mRNA targets. This resulted in significant transcriptional repression of 1605 transcripts. A combinatorial approach using Meg01 mRNA expression profiles following miR-1225-3p overexpression, a computational prediction analysis of miRNA target sequences and a previously published set of differentially expressed platelet transcripts from SCD patients, identified three novel platelet mRNA targets: PBXIP1, PLAGL2 and PHF20L1.

**Conclusions:**

We have identified significant differences in functionally active platelet miRNAs in patients with SCD as compared to controls. These data provide an important inventory of differentially expressed miRNAs in SCD patients and an experimental framework for future studies of miRNAs as regulators of biological pathways in platelets.

## Introduction

Sickle cell disease (SCD) is characterized by chronic hemolytic anemia as a result of a single point mutation in the beta-globin gene (Val–Glu) leading to the formation of a sickle hemoglobin (HbS). This HbS molecule has a propensity to polymerize when deoxygenated, rendering the sickle red blood cells (SS RBCs) less deformable with propensity to cause vaso-occlusion in the microvessels. Central in SCD pathogenesis is acute and chronic endothelial injury and inflammation leading to vasculopathy and activation of the coagulation system. Among the many components of the hemostatic system, increased platelet activation plays a catalytic role in SCD vasculopathy [1], [2], [3]. Markers of platelet activation such as a) increased expression of activation dependent antigens, P-selectin and glycoprotein IIbIIIa, on circulating platelets [3], [4], [5], b) increased plasma concentrations of platelet factor 4 [4], beta-thromboglobulin [4], thrombospondin-1 (TSP-1) [6], [7] and soluble CD40 ligand [8] and c) increased numbers of circulating platelet microparticles [3] have been detected in patients with SCD in steady state and are amplified during acute vaso-occlusive crisis [3]. Activated platelets contribute to vaso-occlusive crises and intimal damage by increased adhesion of SS RBCs to the endothelium [9] via secretion of fibrinogen, von Willebrand factor (vWF) [1] and TSP-1 [7], [10]. In addition, activated platelets play a key role in promoting intimal hyperplasia by secreting vasoactive and mitogenic substances for the fibroblasts and smooth muscle cells such as platelet-derived growth factor (PDGF) and transforming growth factor-beta (TGF-β) [11], [12].

Platelets synthesize an elaborate set of proteins in a timely and signal-dependent manner but little is known as to how their transcriptome is modulated. Gene expression studies of platelets were traditionally limited by the low abundance of platelet RNA which stipulates processing of large volumes of blood (∼50 ml) to obtain 1 to 4 µg of RNA. The development of microarray technology paired with RNA amplification techniques has allowed for high-throughput transcript profiling of platelets in multiple disease states including coronary heart disease [13], essential thrombocythemia [14] and systemic lupus erythematosus [15]. We have successfully analyzed the amplified platelet transcriptome in SCD patients from single donors using these techniques [16] and we identified ∼100 differentially expressed genes in SCD as compared to controls with increased expression of genes involved in arginine metabolism and redox hemostasis. This study indicates that platelets from SCD patients have distinct gene expression patterns possibly involved in SCD-specific platelet biology.

One of the significant advances in molecular biology in the past decade has been the recognition of microRNAs (miRNAs) which are small noncoding RNAs of approximately 18–25 nucleotides in length. They regulate target mRNAs by repressing translation or inducing nucleolytic cleavage through their binding to their 3′ untranslated regions (3′ UTR) [17], [18]. More than 800 miRNAs have been discovered in humans, although the function of many remains unknown. There is evidence that at least one third of the human genome is regulated by miRNAs, and perhaps more than 60% of the human protein-coding genes [18]. Recent data suggest an important regulatory role of miRNAs in hematopoietic differentiation such as megakaryocytopoiesis [19], erythropoiesis [20], [21], and in hematological malignancies [22], [23]. There are very few published studies to date documenting presence of miRNAs in platelets from pooled apheresis samples [24] and from healthy donors [25], [26], [27], [28], [29]. The identity and expression levels of miRNAs in platelets of patients with SCD have not been examined to date.

This is the first comprehensive study undertaken to elucidate the signature miRNAs of platelets from SCD and healthy controls using microarrays in two distinct patient cohorts. Our findings confirm the previous studies showing that anucleate platelets contain diverse and abundant miRNAs. Importantly, we have identified SCD-specific alterations in miRNAs that relate to the SCD platelet transcriptome profile as described in our previously published study [16]. We have further identified three novel target mRNAs as a result of upregulation of miRNA-1225-3p in SCD patients. These findings provide an experimental framework for the study of dysregulation of platelet miRNA and the pathogenesis of SCD.

## Materials and Methods

### Ethics Statement

The study was approved by the Institutional Review Boards of University of Pittsburgh Medical Center (UPMC) and National Heart, Lung, and Blood Institute (NHLBI) and written informed consent was obtained from all participants in accordance with the Declaration of Helsinki.

### Subjects

For the miRNA array study, a cohort of 19 patients with SCD (hemoglobin SS) and 10 controls were selected from the outpatient clinic at NHLBI. To validate the findings of the microarray data, a validation cohort consisting of 7 SCD patients and 7 controls were selected from UPMC. The controls did not have any hemoglobinopathy and individuals on anticoagulant or anti-platelet medications were excluded. We also excluded history of coronary artery disease or its equivalents, atrial fibrillation or infectious disease which could influence platelet reactivity. Smoking history was not available in all our subjects and thus its effect on platelet miRNA expression profile in this study population could not be assessed.

### Platelet Preparation

Twenty milliliters of peripheral blood was collected from patients and controls in CPT tubes and samples were processed within 15 minutes of blood collection. Samples were centrifuged at 150×g for 10 minutes, and platelet-rich plasma was carefully aspirated and re-centrifuged at 150×g for 5 minutes to remove remaining red and white cells. Platelet-rich plasma was centrifuged again at 1500×g for 10 minutes to pellet the platelets. The cell pellet was then washed twice with erythrocyte lysis buffer to remove traces of contaminating red blood cells and once with phosphate-buffered saline (PBS).

Purity of the preparation was checked by flow cytometric measurement of cells expressing glycoprotein IIb/IIIa antigen. In brief, 100 µl of cell preparation was mixed with 100 µl of PBS containing 2% fetal calf serum (FCS). Samples were incubated on ice in the dark for 15 minutes with either fluorescein isothiocyanate (FITC) labeled CD45 antibody to assess leukocyte contamination or phycoerythrin (PE) labeled CD41a antibody for platelets. Mouse anti-human IgG1k-PE and IgG1k-FITC were used as isotype controls (Becton Dickinson, San Jose, Calif). Following incubation, the cells were washed with PBS/FCS, pelleted by centrifugation at 300×g for 5minutes and resuspended in 0.5 ml PBS/FCS. Platelets were identified using BD FACS caliber flow cytometer by their characteristic light scatter and the platelet-specific antibody CD41a binding to glycoprotein IIb/IIIa. At least 25,000 events were counted with fluorescence intensity greater than a threshold set at 1% from the respective negative control sample. In addition, cell count was used to determine purity of the preparation and was checked in a Cell-Dyn Coulter counter 3700 (Abbott Diagnostics, Abbott Park, Ill). For this the platelet pellet was washed with PBS.

### Cell Culture

MEG-01 cells (CRL-2021; American Type Culture Collection [ATCC], Manassas, VA), were used for miRNA transfection experiments. These were cultured in RPMI 1640 medium (ATCC) supplemented with 10% fetal bovine serum and penicillin/streptomycin in a humidified incubator under 5% CO2 at 37°C.

### Transfection of MEG-01 Cells with miR-1225-3p

MEG-01 cells (1×10^6^ ) were transfected with either 200 nM of pre-miRNA 1225-3p or negative control pre-miR™ miRNA ds-oligo precursors (Ambion, Austin, TX) using Amaxa® Nucleofector Kit C (Lonza) and NucleofectorII ® Device (Amaxa Inc. Walkersville, MD) as per manufacturer’s instructions. Cells were incubated for 24 hours with the miRNA prior to RNA purification for gene expression analysis. Transfection efficiency was estimated in parallel by co-transfection of a GFP-expressing plasmid (pmaxGFP® Vector, Lonza) and flow cytometric analysis of GFP-expression 24 hours post Nucleofection®. The pre-miR™-miRNA precursor molecules acting as negative control #1 (Ambion Inc., TX) have a random sequence and have no known human mRNA target.

### RNA Isolation

Platelets and MEG-01 cells were lysed in lysis buffer containing phenol and guanidinium thiocyanate. Total RNA, including miRNAs and mRNAs, was isolated using miRNeasy mini kit (Qiagen, Valencia, CA) and RNAqueous miRNA isolation kit (Ambion, Austin, Texas) at the UPMC and NHLBI sampling locations, respectively. The concentration of the isolated RNA was determined using Nanodrop ND-1000 spectrophotometer (Nanodrop Technologies, Wilmington, Del). Quality and integrity of the total RNA isolated were assessed using the RNA 6000 Pico kit with the Agilent 2100 Bioanalyzer (Agilent Technologies, Palo Alto, CA). The total RNA yield from 20 ml of peripheral blood was in the range of 500–1000 ng.

### Microarrays

MiRNA profiling was performed as previously described by us [30], with an 8×15 K Agilent human miRNA microarray (Version 3.0, Agilent Technologies) containing 866 miRNAs (Sanger miRbase release 9.1), with each miRNA having three or four unique probes on the array. Labeling and hybridization of 100 ng of total RNA was performed according to the protocol described by the manufacturer. Briefly, 100 ng of total RNA was dephosphorylated using calf intestine alkaline phosphatase, denatured with DMSO, and labeled with Cyanine 3 (Cy3) using T4 RNA ligase at 16°C for 2 h. The labeled RNA was purified using Micro Bio-spin 6 columns and hybridized onto the miRNA microarrays at 55°C for 20 h.

Gene expression microarray was performed as previously described by us [30]. Briefly, 100 ng of total RNA was labeled using the One-Color, Agilent Low Input Quick Amp Labeling Kit (Agilent Technologies) following the protocol described by the manufacturer. Briefly, total RNA was used as a template for double-stranded cDNA synthesis. The cDNA was used by T7 RNA polymerase as a template to generate Cy3-labeled cRNA that was used for hybridization on Agilent Sure Print G3 Human GE 8×60 K Microarrays (Agilent Technologies) at 65°C for 17 h.

The arrays were washed with Gene Expression Wash Buffers 1 and 2 (Agilent Technologies) and scanned using the Agilent Microarray Scanner (Agilent Technologies). The scanned images were processed by Agilent’s Feature Extraction software version 9.5.3. All microarray data has been deposited in the Gene Expression Omnibus (GEO) under accession number GSE41575.

### Microarray Data Processing and Analysis

MiRNA microarray data were log base 2 transformed and quantile normalized. Only those miRNAs with mean expression values for each probe greater than 95% of the mean values of the negative control probes under at least one condition, sickle cell disease or control, were considered for further statistical analysis. Gene expression microarray data were log 2-transformed and cyclic loess normalized as previously described by us [30]. The normalized data was expressed as the difference of log of g Processed Signal (Agilent Feature Extraction) and log of geometric mean of controls.

Transformed data from all the arrays were subjected to a principal component analysis to detect outliers. Student’s unpaired t-test was used to identify those miRNAs that were differentially expressed (P<0.05 or <0.1, fold-change>2 (FC>2)) between SCD and controls and disease subgroups using Genespring software (www.agilent.com/chem/genespring) and BRB-Array Tools v.4.1.0. Similar criteria were used to find differentially expressed genes between miR-1225-3p and negative control miRNA (scrambled) transfected MEG-01 cells. The false discovery rate (FDR) was controlled at 5% to correct for multiple comparisons. Microarray data from a previously published platelet gene expression study (NHLBI SCD study) (Gladwin et al, unpublished data) [16] was also reanalyzed using the above criteria. Data visualization and Clustering was performed using Scoregene package as described [31], TreeView [32] and Genomica programs (http://genomica.weizmann.ac.il/).

Multiple algorithms (Targetscan [33]- http://www.targetscan.org, miRanda [34]- http://www.microrna.org and PicTar -http://pictar.org) were then used to refine those miRNAs with relatively platelet-specific targets for validation. To evaluate whether a given pathway was overrepresented in the list of differentially expressed miRNAs and genes, statistical softwares-Ingenuity® Systems (IPA, www.ingenuity.com ) and DAVID Bioinformatics Resources 6.7 [35] were used. DAVID software was also used to look for gene-enrichment associated with a certain biological process (annotation term) from the calculated Fisher Exact p-Value. An EASE score is calculated using the p-values of the individual members of each Functional Annotation Cluster [36]. Smaller p-values and higher scores for a cluster of enriched terms signify more importance of the group in the study. In our study we used a FDR of <0.05 and EASE score of >1.3 as cutoff.

We calculated the enrichment of SCD platelet genes among the differentially expressed genes in the transfected MEG-01 cells by using a hypergeometric distribution test. For the above calculation, we used the total number of genes in the Affymetrix chip (N = 31,355, Affymetrix Human Genome U133 Plus 2.0) and the total number of differentially expressed genes in SCD platelets vs controls (n = 2039 with FDR 5% and n = 518 with FC>2) from the NHLBI SCD study [16] against the differentially expressed genes in the miR-1225-3p transfected MEG-01 cells vs scrambled (x = 2642 with FDR 5% and x = 1037 with FC>2) and the number of genes present in both the lists. An illustration of all the analyses used in this study is presented in **Figure S1**.

### Prediction of microRNA Targets

For the target prediction of the selected miRNAs, we used a novel algorithm, ComiR [37]. By design, ComiR predicts the target genes of a set of miRNA genes by considering multiple miRNA binding events on the same mRNA (in the 3′UTR region). ComiR improves the predictions of four popular motif finders (Targetscan [33], mirSVR [38], PITA [39], miRanda [34]) by weighing their predictions by miRNA expression or by using the Fermi-Dirac model as more accurate prediction of target site occupancy. Then, it integrates the predictions obtained from these four target prediction tools, using a support vector machine (SVM) trained on Drosophila Ago1 immunoprecipitation data. Each gene is assigned with a ComiR score, which is the class probability estimate of the SVM for the gene to be a functional target of the set of miRNAs. ComiR algorithm takes into account for the miRNA abundance levels. Thus, for the same set of miRNAs, different miRNA expression profiles produce different ComiR scores.

### Validation of microRNA and mRNA Data by Real-time Quantitative Reverse Transcription-Polymerase Chain Reaction (qRT-PCR)

To validate the miRNA array data, 10 ng of total RNA was reverse transcribed using TaqMan microRNA Reverse Transcription Kit (Applied Biosystems, Foster City, CA). Real-time PCR (RT-PCR) was performed in triplicate per sample using TaqMan miRNA assays and Taqman Universal PCR Master Mix (Applied Biosystems). PCR reactions were set up to have 0.9 ng of cDNA and were run in ABI 7900 HT real-time PCR system according to the manufacturer’s instructions. The results were analyzed by the comparative cycle threshold method (ΔΔCt method) using RNU43 amplification as endogenous control. Statistical significance was determined by Student t test, setting P<0.05 as threshold.

For mRNA qRT-PCR, total RNA was reverse transcribed to cDNA using High-Capacity cDNA Reverse Transcription kit (Applied Biosystems). RT-PCR was performed on 40 ng of cDNA per reaction using TagMan Gene Expression Assays (Applied Biosystems) (see below) and TaqMan Gene Expression Master Mix (Applied Biosystems). The results were analyzed by the comparative cycle threshold method (ΔΔCt method) using BGUS amplification as endogenous control. Statistical significance was determined by Student t-test, setting P<0.05 as threshold.

TaqMan Assays: MT1X (Hs00745167_sH), IFI6 (Hs00242571_m1), PTPN6 (Hs00169359_m1), FCER1G (Hs00175408_m1), RAP2A (Hs00702699_s1), BCR (Hs01036532_m1), FRMD3 (Hs00604157_m1), IGF1R (Hs00609566_m1), PTGER3 (Hs00168755_m1), PBXIP1 (Hs00933587_m1), PLAGL2 (Hs01122758_m1) and PHF20L1 (Hs01036397_m1), GUSB (Hs99999908_m1).

## Results

### Patient Characteristics

The clinical characteristics of the miRNA array cohort (NHLBI) and the validation cohort (UPMC) are shown in **Table 1** **.** Individuals in the NHLBI cohort consisted of 19 SCD patients and 10 controls. The median age of the patients was 51 years whereas that of controls was 43 years. There was an almost equal distribution of females in both the groups (∼70%). Doppler-estimated increases in pulmonary artery systolic pressure, i.e. elevated tricuspid regurgitant velocity (TRV), was present in 68% of patients and 53% were on Hydroxyurea (HU) at the time of study enrollment. The UPMC cohort consisted of 7 SCD patients and 7 control subjects with a similar median age of ∼38 years. Women were overrepresented in the control group (57%) as compared to patients (29%). Similar to the NHLBI cohort, there were 71% patients with elevated TRV and 57% on HU. The two independent cohorts of SCD patients from UPMC and NHLBI had similar clinical features and no significant differences in the laboratory parameters. Similar numbers of patients in both cohorts were on hemodialysis, chronic transfusion and on Aspirin/Plavix. Subgroup analysis to study the impact of these factors on platelet expression did not yield any significant results.

**Table 1:** Characteristics of Study Participants.*

	NHLBI Cohort Median (Range)		UPMC Cohort Median (Range)		Overall Median (Range)	
Characteristic	SS (N = 19)	Control(N = 10)	SS (N = 7)	Control(N = 7)	SS (N = 26)	Control (N = 17)
Age, y	51 (23–79)	43 (28–61)	38 (22–65)	37 (32–62)	49 (22–79)	40 (28–62)
Women, N (%)	14 (74)	7 (70)	2 (29)	4 (57)	16 (62)	11 (65)
HbSS, %	100		100		100	
Pulmonary Hypertension, N (%)	13 (68)		5 (71)		18 (69)	
Hydroxyurea, N (%)	10 (53)		4 (57)		14 (54)	
Hemoglobin, g/dL	9.3 (6.2–13.2)		7.8 (6.1–10.3)		8.9 (6.1–13.2)	
Platelet count, ×10^−3^/µL	325 (92–943)		215 (102–442)		322 (92–943)	
White blood cell count, ×10^−3^/µL	6.0 (2.3–12.5)		10.4 (5.8–14.4)		6.6 (2.3–14.4)	
Reticulocyte count, ×10^−3^/µL	0.13 (0.04–0.46)		0.15 (0.01–0.49)		0.14 (0.01–0.49)	
Fetal hemoglobin, %	9.9 (1.4–33.8)		2.9 (1.6–9.8)		8.4 (1.4–33.8)	
Lactate dehydrogenase, U/L	291 (192–566)		347 (212–904)		305 (192–904)	
Total bilirubin, mg/dL	1.5 (0.4–7.1)		2.0 (0.6–9.2)		1.7 (0.4–9.2)	

* Age and Laboratory values indicate median (range).

### MicroRNAs are Differentially Expressed in Platelets in Sickle Cell Disease

**Figure 1:**
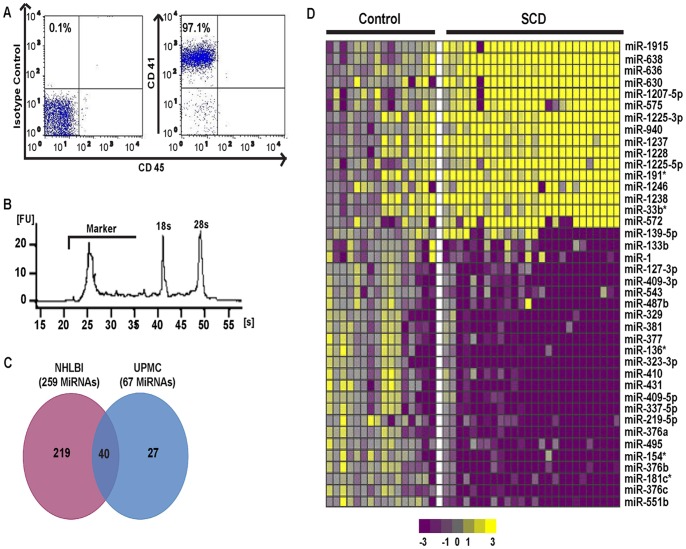
MiRNAs in platelets of SCD patients are differentially expressed. (**A**) Characterization of purified platelet preparation by flow cytometry. (**B**) Bioanalyzer assessment of RNA samples from purified platelet preps showing good quality with RIN of 7.8. (**C**) Venn diagram comparing differentially expressed miRNAs from two independent study cohorts. Comparison of 259 differentially expressed miRNAs from NHLBI samples and 67 differentially expressed miRNAs from UPMC samples. In total, there are 40 miRNAs overlapping between the two cohorts. (**D**) The heatmap depicts the 40 statistically significant (*p*-value<0.05, FC>2) differentially expressed miRNAs between SCD and controls that were common between the two cohorts. Columns represent individual samples and each represents a miRNA. Upregulated miRNAs expression levels are shown in progressively brighter shades of yellow, depending on the fold difference. Downregulated miRNAs are shown in progressively brighter shades of purple. No difference is represented as grey. The names of the miRNAs are displayed to the right of the heatmap.

Our protocol was optimized to obtain a highly pure platelet population with minimum leukocyte contamination. For each sample, flow cytometric assessment for platelet purity revealed a highly enriched platelet population with more than 95% cells positive for CD41a, with less than 0.2% CD45+ leukocytes and non-CD41a, non-CD45 cellular debris (**Figure 1A**). Cell counts on the platelet preparations demonstrated that the white blood cell count ranged from 0–0.3×10^∧3^/µl and platelets ranged from 1–2×10^∧6^/µl. This amounted to roughly 0.03% leukocytes or less in the platelet preparations. Platelet gene expression profiling studies of platelet rich plasma have been considered specific with contaminating leukocytes of only 0.4% [25], [27], [40], [41]. In addition, we performed qRT-PCR in a few SCD and control platelet samples to assess the expression of residual leukocyte-specific message (CD45/PTPRC). We did not find any significant difference in the expression level of CD45 between SCD and control samples (**Figure S3**). This suggests that any residual leukocyte contamination is present in all the samples in equal degrees and can be treated as ubiquitous background that won’t affect the profiles of the differentially expressed platelet miRNAs. Following RNA isolation and quality control using the Bioanalyzer, RNA samples with RIN value>7 were selected for further microarray analysis (**Figure 1B**).

We compared the expression profiles of platelet human miRNAs in both cohorts using the Agilent miRNA platform. Stringent statistical filters of p-value of <0.05 and FC>2 were applied and the differentially expressed miRNAs between SCD patients and controls were determined separately for the UPMC and NHLBI cohorts. Our final differentially expressed list included only common miRNAs identified in both cohorts, increasing the selection stringency on our results. The larger NHLBI cohort revealed 259 differentially expressed miRNAs passing our statistical filters and the UPMC cohort identified 67 differentially expressed miRNAs between SCD patients and controls. Forty miRNAs were identified with significantly different levels in platelets from SCD patients in both cohorts (**Figure 1C**). The pattern of platelet miRNA expression is presented as a heatmap using Scoregene which classifies the samples based on their similarity (**Figure 1D**). Out of these 40 differentially expressed miRNAs 16 were upregulated and 24 were downregulated in SCD, consistently in both cohorts (**Figure S2**). The 20 most significant differentially expressed miRNAs are listed in **Table 2** in rank order of their expression level.

**Table 2:** Twenty most differentially expressed (A) up- and (B) down-regulated microRNAs (P<0.05, fold change>2) between sickle cell disease (N = 26) and controls (N = 17) ranked according to p-value (low to high).

(A)	microRNA	P value	FoldChange	Chromosome
	miR-638	6.5×10^−6^	15.9	19p13.2
	miR-940	3.2×10^−5^	14.9	16p13.3
	miR-636	6.5×10^−5^	6.4	17q25.1
	miR-1238	1.1×10^−4^	10.9	19
	miR-1225-3p	1.1×10^−4^	10.3	16p13.3
	miR-1207-5p	1.1×10^−4^	13.5	8
	miR-33b*	1.1×10^−4^	7	17p11.2
	miR-1228	1.3×10^−4^	12	12
	miR-630	1.6×10^−4^	24.2	15q24.1
	miR-191*	2.2×10^−4^	10.3	3p21.31
**(B)**	**microRNA**	**P value**	**Fold** **change**	**Chromosome**
	miR-376a	6.5×10^−6^	11.7	14q32.31
	miR-381	6.5×10^−6^	6.8	14q32.31
	miR-409-3p	6.5×10^−6^	6.5	14q32.31
	miR-377	6.5×10^−6^	6.5	14q32.31
	miR-376c	1.6×10^−5^	12.1	14q32.31
	miR-495	1.8×10^−5^	4.5	14q32.31
	miR-376b	2.3×10^−5^	4.5	14q32.31
	miR-181c*	2.3×10^−5^	3.9	19p13.13
	miR-487b	3.2×10^−5^	6.3	14q32.31
	miR-337-5p	3.2×10^−5^	4.6	14q32.2

### Computational Prediction of microRNA Targets with ComiR

As an initial evaluation of these 40 differentially expressed miRNAs, a bioinformatics-based data mining approach was used. Our newly developed algorithm, ComiR [37], was used in this analysis. Based on the miRNA expression profiles of the 40 differentially expressed miRNAs in the 43 subject samples the ComiR likelihood score was calculated for each mRNA in each of the 43 samples. This score corresponds to the likelihood that the mRNA expression is affected by the set of the 40 miRNAs expressed at the sample associated miRNA abundance levels. Then for each sample, a rank-order list of all mRNAs was created based on the ComiR probability that their expression was affected by the set of 40 miRNAs. To compare the rank of each mRNA obtained from the SCD samples with that from the controls, we performed a non-parametric Wilcoxon rank-sum test since the population was not normally distributed and further applied FDR of 5% for multiple comparisons correction. In order to select the genes differentially predicted as target of the miRNAs expressed in the two groups of samples, we chose those associated with a p-value lower than 0.05 and which were also included in the top 10% predicted targets by ComiR in at least one of the two groups of samples. The second filter was introduced to remove all the genes with a significantly different rank in the two groups that were not predicted as targets in any of the two groups. In this way, we ended up with a list of 2309 genes. To determine whether the ComiR generated list of 2309 target genes was reflecting gene expression changes in SCD platelets, we compared it against the list of differentially expressed genes from the NHLBI SCD study (N = 518, FDR 5% and FC 2).We found 89 genes present in both datasets which was more than expected by chance as determined by hypergeometric distribution test (p-value 3.39497E−14). Our results suggest that ComiR provides a meaningful set of putative targets that may be regulated by the 40 differentially expressed miRNAs in platelets (**Table S1**).

### Downregulated microRNAs from miR-329, miR-376 and miR-154 Families in Platelets from Sickle Cell Disease Map to the Maternally Imprinted 14q32.31 Locus

Amongst the 24 significantly downregulated miRNAs in SCD platelets, 14 were found to be members of three families - miR-329, miR-376 and miR-154 (**Table 3**). These miRNAs are transcribed from the genes located at the 14q32.31 locus in the human genome and map within a ∼40 kb interval. Since the majority (∼60%) of the downregulated miRNAs that were identified in SCD platelets belong to these families, we speculated that these miRNAs and their targets may have an important role in platelet physiology and SCD. Using Targetscan we identified a total of 3583 conserved targets of all these 14 miRNAs. A Functional Annotation Clustering of these genes using DAVID Bioinformatics Resources 6.7 revealed that a significant number of them are involved in the regulation of transcription, regulation of cellular biosynthetic processes, cell morphogenesis and motility (**Table 4**). We used KEGG charts from DAVID to explore the distribution of these target genes among various biological pathways and observed that all three miRNA families are associated with signaling pathways such as Wnt signaling, TGF-β signaling and adherens junction. **Table 5** shows the KEGG pathways that are highly enriched among all the miRNAs of the 3 miRNA families in our study, after using a FDR of 5% and EASE score of >1.3 as cutoff.

**Table 3:** Three distinct microRNA families among the differentially expressed microRNAs.

miR Family	microRNAs	Regulation	FoldChange	p-value
**hsa-miR-154**	miR-381	down	6.8	6.49E−06
	miR-409-3p	down	6.5	6.49E−06
	miR-377	down	6.5	6.49E−06
	miR-487b	down	6.3	3.17E−05
	miR-323-3p	down	2.7	3.17E−05
	miR-410	down	4.8	1.08E−04
	miR-409-5p	down	2.9	1.26E−04
	miR-154*	down	2.8	1.38E−04
**hsa-miR-329**	miR-495	down	4.5	1.78E−05
	miR-329	down	2.4	6.34E−04
	miR-543	down	2.8	0.002
**hsa-miR-376**	miR-376a	down	11.7	6.49E−06
	miR-376c	down	12.1	1.62E−05
	miR-376b	down	4.5	2.26E−05

**Table 4:** Functional annotation clustering of predicted target genes to the differentially expressed microRNAs belonging to three distinct families located at chromosome 14q32.31.

Cluster	EASE score	Count (%)	p-value	Fold enrichment
Regulation of transcription	18.7	750 (21)	1.20E−31	1.4
Positive regulation of RNA metabolic process	14.5	198 (5.5)	3.95E−24	2.1
Negative regulation of gene expression	10.2	191 (5.3)	3.26E−18	1.9
Cellular component morphogenesis	7.5	151 (4.2)	4.78E−14	1.9
Cell migration	5.5	106 (3)	2.71E−09	1.9

**Table 5:** Top enriched pathways regulated by predicted target genes of miRNAs belonging to 154, 329 and 376 families (FDR<0.05).

Pathway	Count	p-value	Foldenrichment
Wnt signaling pathway	70	6.99E−11	2.4
Pathways in cancer	116	2.82E−09	1.8
Adherens junction	42	2.53E−08	2.8
TGF-beta signaling pathway	40	4.66E−05	2.4
Ubiquitin mediated proteolysis	53	2.34E−04	2.0
Axon guidance	50	5.17E−04	2.0
Tight junction	51	7.44E−04	2.0

### Computational Evaluation of Differentially Expressed microRNAs and Validation of Microarray Data

**Figure 2:**
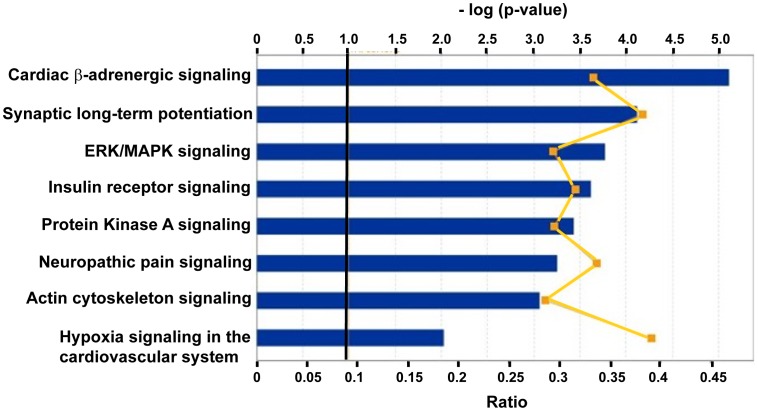
Most significant predicted canonical pathways regulated by the target genes of the 3 miRNAs: 376a, 409-3p and 1225-3p (p-value<0.01). Orange squares depict ratio which is the number of target genes associated with each pathway. The vertical line across the bars represents the cut-off of the p-value.

**Figure 3:**
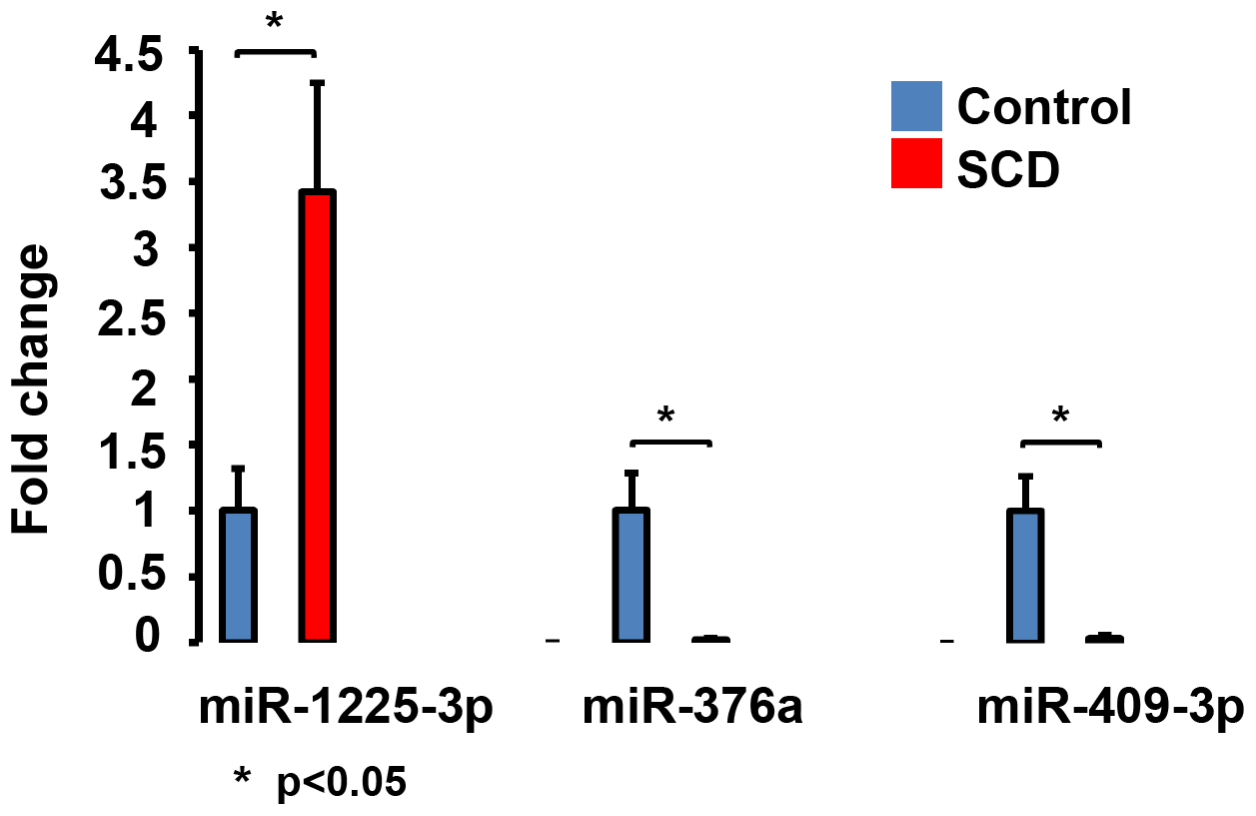
miR-376a and miR-409-3p are downregulated and miR-1225-3p is upregulated in SCD platelets. qRT-PCR validation of the microarray data. For each miRNA the blue bar represents controls (n = 26) and the red bar represents SCD (n = 17). Y-axis shows fold change of miRNAs in SCD samples with the expression level in controls set to 1. Error Bars represent standard deviation.

For the purpose of validating the microarray results, the expression levels of several miRNAs were validated by qRT-PCR. Limited by the quantity of RNA per sample we had to restrict ourselves to choosing miR-376a of the miR-376 family, miR-409-3p of the miR-154 family and miR-1225-3p of the most upregulated miRNAs. These three miRNAs were selected based on the following criteria 1) miRNA target databases (TargetScan, miRanda, and PicTar) predicted targets to these miRNAs which were relevant to platelet biology. Gene ontology analysis revealed that predicted targets of these 3 miRNAs were enriched for regulation of transcription, transcription factor activity and regulation of RNA metabolic process. 2) ingenuity pathway analysis of the predicted targets revealed top canonical pathways (as shown in Figure 2) that have been previously identified in relation to megakaryocyte/platelet signaling such as beta-adrenergic signaling, Protein Kinase A signaling, ERK/MAPK signaling, insulin receptor signaling and regulation of the actin cytoskeleton (reviewed in [42], [43], [44], [45], [46]) (**Figure 2**). These three miRNAs were also amongst the top twenty most differentially expressed between SCD vs controls (**Table 2**). qRT-PCR was performed in control and SCD samples of both cohorts and confirmed that miR-376a and miR-409-3p were significantly lower while miR-1225-3p was significantly higher in SCD as compared to control samples (**Figure 3**).

### MicroRNA-mRNA Regulation in Megakaryocytic Cell Line

To explore the functional role of miR-376a, 409-3p and miR-1225-3p we used MEG-01 cell line in the place of human megakaryocytes, which are difficult to obtain in large numbers. To mimic the downregulated state of miR-376a and 409-3p in platelets, we needed to transfect MEG-01 cells with inhibitors of these miRNAs. Unfortunately, our initial PCR studies showed that the baseline levels of miR-376a and miR-409-3p in MEG-01 cells were already very low and often undetectable, making this approach unfeasible. On the other hand, we could determine the targets of miR-1225-3p by replicating the upregulated state in SCD platelets by transfecting MEG-01 cells with a miR-1225-3p mimic. Our hypothesis was that the overexpressed miRNAs would cause mRNA degradation by directly binding to the 3′UTR of its targets and we would be able to identify these potential targets by a negative correlation between the miRNA and the differentially expressed mRNAs.

**Figure 4:**
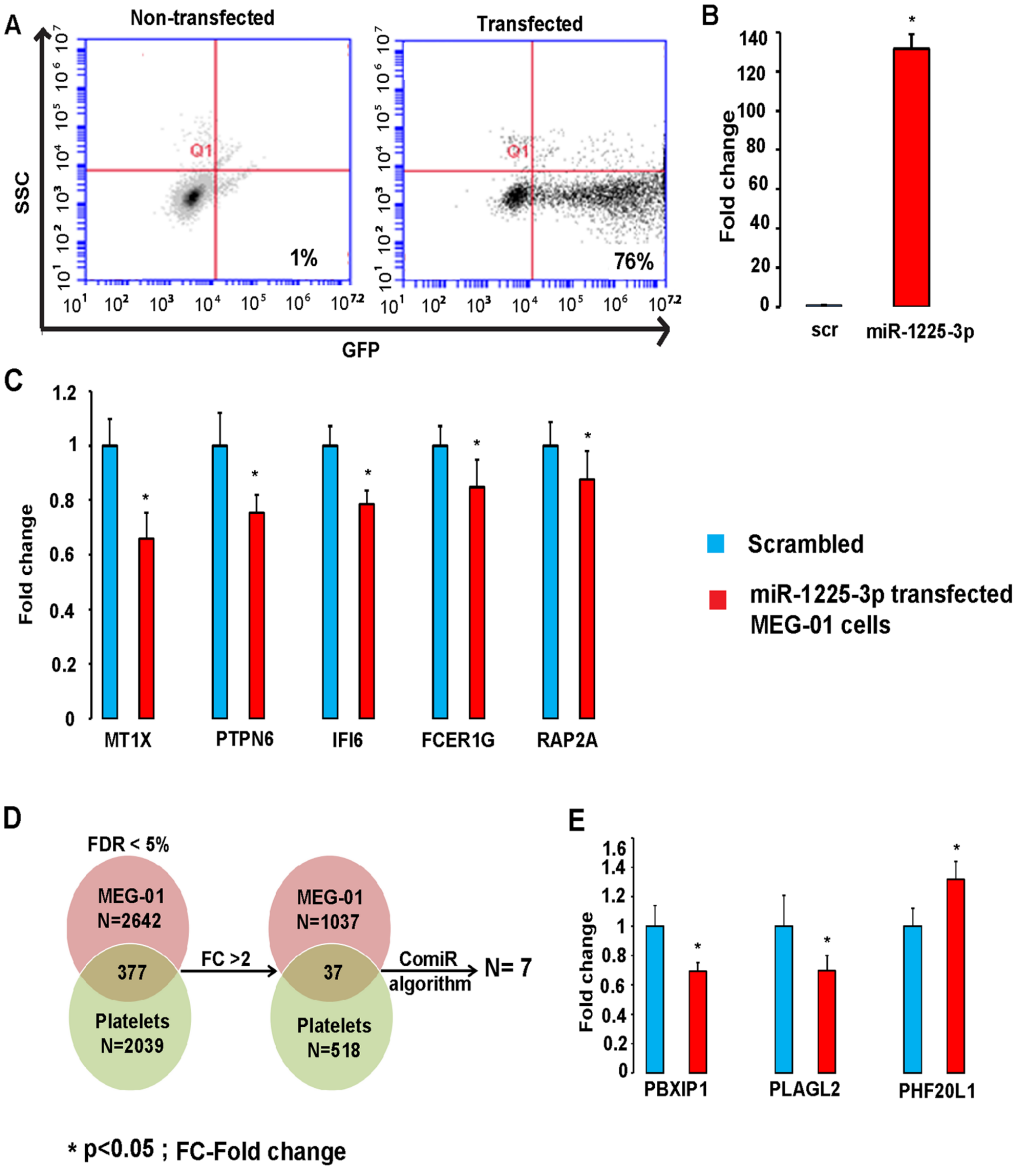
miR-1225-3p regulates gene expression after transfection in MEG-01 cells. (**A**) Expression of the flourescent marker pmaxGFP plasmid as determined by flow cytometry demonstrates 76% GFP expressing cells out of the viable cell population. (**B**) qRT-PCR assay confirms overexpression of miR-1225-3p in MEG-01 cells (n = 5) vs scrambled (scr) (n = 5). (**C**) MT1X, PTPN6, IFI6, FCER1G and RAP2A are significantly downregulated in MEG-01 transfected cells as validated by qRT-PCR. (**D**) Schematic representation of overlap in differentially expressed genes between miR-1225-3p transfected MEG-01 cells and platelets from SCD patients. The numbers in the circles denote the differentially expressed genes with the leftmost figure representing genes using a FDR of 5% and the rightmost figure after applying a stringent statistical filter of FC>2. Further overlap with ComiR predicted target list of the 40 differentially expressed miRNAs resulted in a list of 7 genes potentially regulated by miR-1225-3p. (**E**) Out of the 7 genes, PLAGL2 and PBXIP1 genes are significantly downregulated and PHF20L1 is significantly upregulated in miR-1225-3p transfected cells vs scrambled. In the qRT-PCR figures (B, C and D), for each gene, the blue bar represents the cells transfected with scrambled RNA (n = 5) and the red bar represents the miR-1225-3p transfected cells (n = 5). Y-axis shows fold change of miR-1225-3p in transfected cells with the expression level in scrambled set to 1. Error bars are based on standard deviation. *denotes p-value<0.05.

Successful transfection of MEG-01 cells with miR-1225-3p mimic was confirmed by GFP detection and qRT-PCR (P<0.05, transfected vs scrambled cells, n = 5 in each group) (**Figures 4A and 4B**). A transfection efficiency of 70–75% was achieved (**Figure 4A**). Microarray analysis of miR-1225-3p transfected cells relative to the scrambled identified a total of 2642 genes differentially expressed at a FDR of 5%. Out of these, a total of 1037 genes were upregulated and 1605 genes were downregulated. Out of the 2642 genes, 1037 genes were changed at a FC>2.

In order to decide whether this set of 2642 differentially expressed genes from the transfected cells (FDR 5%) is enriched in platelet-related genes, we interrogated our data against a defined and general set of megakaryocytic and platelet genes. We explored the IPA knowledge base for existing literature on genes annotated under the terms “platelets” and “megakaryocytes”. This generated a list of 426 genes (IPA list) which have been established to be associated with biological functions in platelets and megakaryocytes by a variety of studies. Amongst the 2642 genes a total of 75 genes overlapped with the genes from the IPA list. The calculated probability of finding overrepresentation of platelet and megakaryocyte genes amongst the differentially expressed genes in transfected cells was more than expected by chance as determined by hypergeometric distribution test (p-value 5.28 E-08). This comparison provides additional external validity that miR-1225-3p is a strong candidate for regulating SCD-related platelet biology. A list of these 75 genes with their biological functions pertaining to platelets and megakaryocytes is presented in **Table S2**.

To validate our microarray results, we tested 5 genes (MT1X, RAP2A, IFI6, FCER1G and PTPN6) using qRT-PCR in each of the miR-1225-3p and scrambled transfected samples. All of the 5 genes were downregulated in miR-1225-3p transfected cells according to microarray data and were chosen based on fold change values>3, p-value<0.05 and microarray signal intensity in logarithmic scale were more than the median expression values of all the genes in each sample/array. All five genes were confirmed by qRT-PCR to be downregulated in miR-1225-3p transfected cells (**Figure 4C**).

### Identification of miR-1225-3p Megakaryocyte/Platelet-Specific Target Genes

Although 75 out of 2642 differentially expressed genes are known to be associated with platelet and megakaryocytic biology, we wanted to identify novel genes that may play a role in SCD. For this purpose, we compared the genes that change as a response to miR-1225-3p over-expression (x = 2642) to the list of genes that are differentially expressed in SCD platelets (n = 2039) (NHLBI SCD study). A total of 377 genes were identified to be common between the two datasets after applying a FDR of 5% and the probability of this finding to occur not merely by chance was significant as calculated by the hypergeometric distribution (p-value: 1.82E−53)) (**Figure 4D**). Out of the 377 differentially expressed genes, 37 show a FC>2 with a significant hypergeometric distribution as well (p-value: 6.4E−06) (**Figure 4D**). Notably, 241 out of these 377 common genes were downregulated in both miR-1225-3p transfected MEG-01 and SCD platelets.

Using the list of genes obtained from ComiR prediction algorithm we then sought to find the common genes between the differentially expressed genes from miR-1225-3p transfected MEG-01 cells and platelet samples from NHLBI SCD study (**Figure 4D**). This resulted in a list of 57 genes using a filter of 5%FDR or 7 genes when an additional FC>2 filter was applied (**Table 6**). Of these 7 genes, 3 were downregulated: PBXIP1, PLAGL2 and IGF1R; and 4 were upregulated: BCR, PHF20L1, PTGER3 and FRMD3. qRT-PCR confirmed that PBXIP1, PLAGL2 and PHF20L1, were indeed differentially expressed between transfected and scrambled cells (p-value<0.05) (**Figure 4E**).

**Table 6:** List of novel putative target genes of miR-1225-3p from the differentially expressed genes (FDR<5%, FC>2).

Gene symbol	Gene description	Regulation	Fold Change
BCR	Breakpoint cluster region	Up	9.5
PHF20L1	PHD finger protein 20-like 1	Up	2.5
PTGER3	Prostaglandin E receptor 3 (subtype EP3)	Up	2.2
FRMD3	FERM domain containing 3	Up	2.2
PBXIP1	Pre-B-cell leukemia homeobox interacting protein 1	Down	5.1
PLAGL2	Pleomorphic adenoma gene-like 2	Down	2.7
IGF1R	Insulin-like growth factor 1 receptor	Down	2.2

### Comparison of Platelet microRNAs in Sickle Cell Disease Based on Hydroxyurea Treatment and Tricuspid Regurgitant Jet Velocity

**Figure 5:**
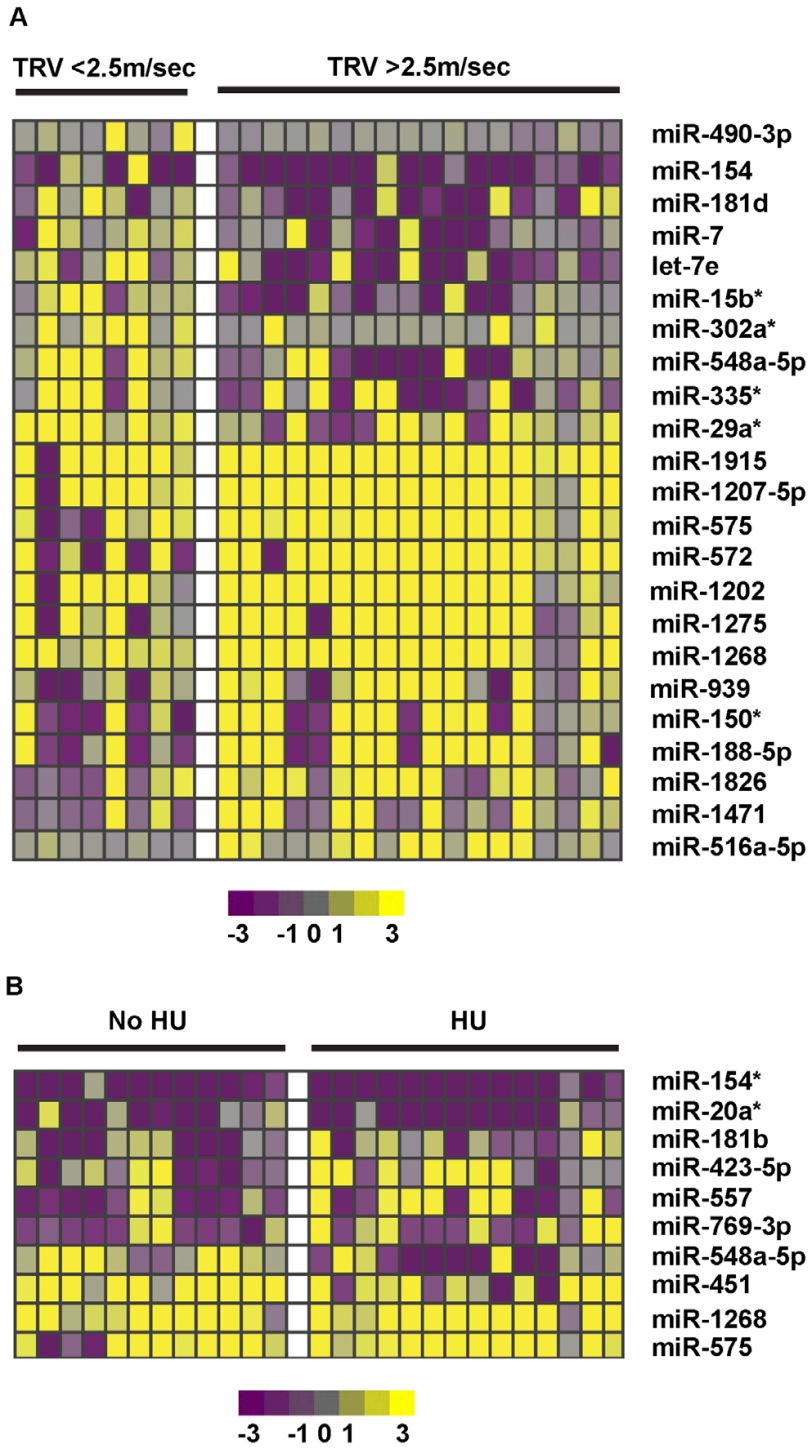
miRNAs in platelets have a unique expression profile in SCD patients based on Doppler-estimated TRV and HU treatment. (A) The heatmap represents 23 statistically significant (*p*-value<0.1, FC>2) differentially expressed miRNAs between subjects with (N = 18) and without (N = 8) elevated TRV (>2.5 m/sec). (B) The heatmap represents 10 statistically significant (*p*-value<0.1, FC>2) differentially expressed miRNAs between the subjects on (N = 14) and off HU (N = 12). Every row represents a gene and every column is a patient. Upregulated miRNAs are shown in yellow color, downregulated miRNAs are shown in purple and no difference is grey. The names of the miRNAs are displayed to the right of heatmap.

We explored miRNA expression changes in subsets of SCD patients based on HU treatment and presence of elevated Doppler-estimated TRV (>2.5 m/sec). We performed a direct comparison between these patient subsets to identify miRNAs that are differentially expressed under each condition. We identified 23 differentially expressed miRNAs between subjects with and without increased TRV and 10 differentially expressed miRNAs between the subjects on and off HU (**Figures 5A and 5B**). Surprisingly, but consistent with prior studies evaluating global mRNA transcriptional profiles, the miRNA expression profiles of patients on HU treatment was almost identical to those not on HU [**47**]. Similarly, there are only subtle differences in the platelet miRNA expression in patients with and without high TRV.

## Discussion

In this study, we identify a complex population of miRNAs in circulating platelets in SCD as well as healthy individuals. Our findings indicate that, platelets from SCD patients in two independent cohorts had a dramatic and significant difference in the expression pattern of 40 miRNAs, suggesting that dysregulation of platelet miRNAs are implicated in the pathogenesis of SCD. Stringent criteria were applied for the purification of platelets from whole blood samples to ensure that the observed miRNA profiles reflect changes occurring in platelets and not in other blood cell types. In addition, we found the expression of the 40 differentially expressed miRNAs from our study to be undetectable in the miRNA profile of PBMCs [6], thus providing an additional validation that platelets have a distinct expression pattern. Although all the differentially expressed miRNAs could play an important role in SCD platelet biology, we chose to focus on miR-1225-3p which was found to be upregulated in SCD platelets since it proved to have a considerable number of predicted targets with potential biologic role in platelets. We experimentally demonstrated that dysregulation of miR-1225-3p in a cell culture model related to platelets (Meg-01) had a dramatic impact on gene expression profile and a significant number of the differentially expressed genes (N = 377) were also changed in SCD platelets. Furthermore, nearly 18% of the differentially expressed genes from the transfected cells have been experimentally proven to be relevant to megakaryocyte and platelet function (IPA list), providing additional validity for our findings and suggesting a key role for miR-1225-3p in platelet biology and SCD. With the help of the new prediction algorithm, ComiR, we reduced the list of the differentially expressed genes (N = 377) down to 3 genes which could be regulated directly or indirectly by the miR-1225-3p: PBXIP1, PLAGL2 and PHF20L1.

The first target, PBXIP1 (pre-B-cell leukemia homeobox interacting protein) regulates the transcription factor PBX1 by blocking of the DNA binding PBX1-HOX nucleoprotein complexes to their target regulatory elements. PBX proteins have been shown to play an important role as transcription factors for maintenance of hematopoiesis. An example of this has been shown by overexpressing PBXIP1 in CD34+ stem cells and erythroleukemia cell line (K562) causing induction of erythroid colony formation. We have documented low PBXIP1 expression in SCD platelets (NHLBI SCD study) and downregulation in cells overexpressing miR-1225-3p. These findings support a role of miR-1225-3p in regulating PBXIP1 which could affect lineage commitment by promoting megakaryopoiesis and opposing erythropoiesis. Our second target, PLAGL2 (Pleomorphic adenoma gene-like 2),belongs to the PLAG family of zinc-finger transcription factors [48]. PLAGL2 can act as either an oncogene by activating thrombopoietin receptor (Mpl) in hematopoietic progenitors or a tumor suppressor by initiating cell cycle arrest and apoptosis via p73 activation. A significant role of PLAGL2 is the regulation of TNF-α induced expression of NCF2 gene and subsequent p67*phox* protein (cytosolic component of NADPH oxidase) and superoxide production [49]. NADPH oxidase is an important source of reactive oxygen species (ROS) in human platelets and MEG-01 cells. Downregulation of PLAGL2 by miR-1225-3p overexpression in MEG-01 cells and platelets (NHLBI SCD study), may link miR-1225-3p to a compensatory role in limiting oxidative stress. Similarly, analysis of the transcriptome of peripheral blood mononuclear cells in SCD patients revealed significant up-regulation of catalytic anti-oxidant enzyme systems [47]. The third target, PHF20L1 (PHD finger protein 20-like 1), along with its close ortholog PHF20 has been found to be a part of the multisubunit human histone acetyltransferase complex, involved in histone acetylation and post-translational modification [50]. Knockout of PHF20 in mice has been seen to cause defects in many hematopoietic tissues including bone marrow and leads to aberrant thymocyte development [51]. To date very little is known regarding the function of PHF20L1.

An intriguing finding from this study was that of downregulation of members of the miR-154, the miR-329 and miR-376 family in SCD platelets. To our knowledge this is the first time that miRNA families have been associated with a distinct signature in platelets. These miRNA clusters are located at 14q32.31 locus in DLK1-DIO3 genomic region which includes the maternally imprinted genes MEG3, MEG8 and antisense RTL1 [52]. The expression of the maternally expressed as well as repressed imprinted genes of this entire locus is defined by the methylation status of their promoters [53]. Dysregulation of this miRNA cluster has been well-described in relation to hematologic malignancies, suggesting epigenetic regulation of transcription factors affecting development and proliferation of hematopoietic cells [54]. In the context of SCD, further studies exploring the impact of methylation-acetylation changes of this locus on the miRNA expression levels in cells of the megakaryocyte lineage may provide an important clue to the profound clinical heterogeneity observed in this monogenic disorder. With this understanding, therapeutic epigenetic modifications of these miRNA clusters might become a novel approach for controlling the dysregulation of homeostasis caused by platelets in SCD. The miRNAs from the 14q32 region are involved in the regulation of important signaling pathways such as Wnt and TGF-β [30] which have been implicated in platelet function, activation and thrombosis [55], [56]. Among the predicted targets of miRNAs of the 14q32 region, we found members of the adherens and tight junction pathways which have not been previously reported in conjunction with this locus. This novel finding might be relevant to SCD pathophysiology, as these pathways have been shown to be involved in complex platelet-endothelial interactions and their deregulation might play a role in endothelial dysfunction (reviewed in [57], [58]).

It can be assumed that since platelets are anucleate, the biogenesis of the platelet miRNAs is from megakaryocytic precursor cells, in which pri-miRNAs are encoded and converted into pre-miRNAs before platelet formation. Nevertheless, platelets possess a miRNA processing machinery, including Dicer, TRBP2 and Ago2 [25] which enables them to process pre-miRNA into mature miRNA. Both of the above mechanisms contribute to platelet miRNA profiles which suggests that the differential expression of platelet miRNAs in patients with SCD could be a result of (1) increased numbers of short-lived circulating young, transcriptionally active platelets in SCD (megathrombocytes) [59], [60], [61], [62], (2) alterations in the megakaryocyte transcriptome, and (3) alterations in the stability of the pre-formed miRNA in the circulating platelets exposed to various physiological and pathological conditions in SCD.

Undoubtedly there are certain limitations of our study. First, there was a significant difference in the number of differentially expressed miRNAs between the NHLBI and UPMC. This possibly is a result of the phenotypic heterogeneity seen in the SCD population from the two locations. Additionally, the ability to detect larger set of differentially expressed miRNAs between SCD and controls in UPMC cohort might be limited due to the smaller sample size. The RNA preparation methodology was also different between the samples derived from UPMC and NHLBI. To address these concerns, we analyzed the miRNA expression profile in the two cohorts independently and chose only those miRNAs that were different in both cohorts. By doing so, we specifically measure profiles relevant to SCD, as opposed to the confounding factors such as age, gender and coexisting medical conditions. Moreover, the list of differentially expressed platelet miRNAs common to both cohorts, confirms the robustness of this miRNA signature profile over a varied SCD population. Second, we were limited by the amount of blood drawn which limited the quantity of RNA obtained. Therefore, we were able to validate only a few of the differentially expressed miRNAs. Third, although we have identified three novel targets, PBXIP1, PLAGL2 and PHF20L1 linked to the expression of miR-1225-3p, we do not provide evidence of their role in alteration of platelet phenotype and biology. This needs to be further explored in megakaryocytes obtained by differentiating primary cell lines. Last, while we have identified a distinctive downregulation of miRNAs localized to the epigenetically regulated miRNA cluster on chromosome 14q32, we were unable to perform a detailed analysis of the regulation of this region in SCD. Analysis of the epigenomic changes at this cluster in platelets cannot be carried out because of lack of genomic DNA collected in these studies.

This is the first human platelet miRNA profiling study in SCD platelets aiming to understand platelet gene regulation. SCD patients are known to have activated platelets which contribute to their thrombophilic state but the key molecular events and pathways involved are still unknown. There is now evidence regarding the role of miRNAs in hemostasis/thrombosis where certain miRNAs associate with platelet reactivity [27], [28] but very little is known about the mechanistic role of miRNAs in SCD platelets. A fundamental aspect of altered miRNA profile is to affect a number of cellular targets but they could also have important paracrine effects. For instance, microparticles shed from platelets in the circulating blood raises a possibility of them serving as a delivery vehicle for miRNAs to targeted vascular sites [26], [63], [64], [65]. Thus, the change in the miRNA expression profile in SCD could not only modulate intrinsic platelet functions but also influence peripheral blood and vascular cells. Our study provides an important inventory of SCD associated miRNAs which could shed light on the regulatory pathways involved in the pathogenesis of this complex disease and is another step towards this long-sought goal.

## Supporting Information

Figure S1 Workflow for the bioinformatics analyses used in the study for predicting target genes of miR-1225-3p.
(TIF)

Figure S2 Two heatmaps for each of the cohorts-UPMC and NHLBI, depicting the 40 statistically significant (*p*-value<0.05, FC>2) differentially expressed miRNAs between SCD and controls. The two cohorts display consistency in the directionality of the differentially expressed miRNAs between controls and SCD samples. Columns represent individual samples and each represents a miRNA. Upregulated miRNAs expression levels are shown in progressively brighter shades of yellow, depending on the fold difference. Downregulated miRNAs are shown in progressively brighter shades of purple. No difference is represented as grey. The names of the miRNAs are displayed to the right of the heatmap.
(TIF)

Figure S3 Expression of leukocyte-specific CD45/PTPRC by RT-PCR in SCD and control platelet samples did not reveal a significant difference. For each miRNA the blue bar represents controls and the red bar represents SCD. Y-axis shows fold change of miRNAs in SCD samples with the expression level in controls set to 1. Error Bars represent standard deviation.
(TIF)

Table S1 List of 89 common genes between ComiR generated list of 2309 target genes and list of differentially expressed genes from the NHLBI SCD study (N = 572, FDR 5% and FC 2).
(XLSX)

Table S2 List of differentially expressed genes (FDR<5%) in transfected cells vs scrambled cells with functional annotations pertaining to platelet and megakaryocytes (n = 75) based on IPA knowledge base.
(DOCX)

## References

[1] Blann AD, Marwah S, Serjeant G, Bareford D, Wright J (2003) Platelet activation and endothelial cell dysfunction in sickle cell disease is unrelated to reduced antioxidant capacity. Blood Coagul Fibrinolysis 14: 255–259. 12695748 10.1097/01.mbc.0000061293.28953.8c

[2] Villagra J, Shiva S, Hunter LA, Machado RF, Gladwin MT, et al (2007) Platelet activation in patients with sickle disease, hemolysis-associated pulmonary hypertension, and nitric oxide scavenging by cell-free hemoglobin. Blood 110: 2166–2172. 17536019 10.1182/blood-2006-12-061697 PMC1976348

[3] Wun T, Paglieroni T, Rangaswami A, Franklin PH, Welborn J, et al (1998) Platelet activation in patients with sickle cell disease. Br J Haematol 100: 741–749. 9531343 10.1046/j.1365-2141.1998.00627.x

[4] Tomer A, Harker LA, Kasey S, Eckman JR (2001) Thrombogenesis in sickle cell disease. J Lab Clin Med 137: 398–407. 11385360 10.1067/mlc.2001.115450

[5] Mohan JS, Lip GY, Bareford D, Blann AD (2006) Platelet P-selectin and platelet mass, volume and component in sickle cell disease: relationship to genotype. Thromb Res 117: 623–629. 16051315 10.1016/j.thromres.2005.05.010

[6] Browne PV, Mosher DF, Steinberg MH, Hebbel RP (1996) Disturbance of plasma and platelet thrombospondin levels in sickle cell disease. Am J Hematol 51: 296–301. 8602630 10.1002/(SICI)1096-8652(199604)51:4<296::AID-AJH8>3.0.CO;2-R

[7] Novelli EM, Kato GJ, Ragni MV, Zhang Y, Hildesheim ME, et al (2012) Plasma thrombospondin-1 is increased during acute sickle cell vaso-occlusive events and associated with acute chest syndrome, hydroxyurea therapy, and lower hemolytic rates. Am J Hematol 87: 326–330. 22318901 10.1002/ajh.22274 PMC3619659

[8] Lee SP, Ataga KI, Orringer EP, Phillips DR, Parise LV (2006) Biologically active CD40 ligand is elevated in sickle cell anemia: potential role for platelet-mediated inflammation. Arterioscler Thromb Vasc Biol 26: 1626–1631. 16601237 10.1161/01.ATV.0000220374.00602.a2

[9] Antonucci R, Walker R, Herion J, Orringer E (1990) Enhancement of sickle erythrocyte adherence to endothelium by autologous platelets. Am J Hematol 34: 44–48. 2109530 10.1002/ajh.2830340110

[10] Brittain HA, Eckman JR, Swerlick RA, Howard RJ, Wick TM (1993) Thrombospondin from activated platelets promotes sickle erythrocyte adherence to human microvascular endothelium under physiologic flow: a potential role for platelet activation in sickle cell vaso-occlusion. Blood 81: 2137–2143. 8471771

[11] Ross R, Glomset J, Kariya B, Harker L (1974) A platelet-dependent serum factor that stimulates the proliferation of arterial smooth muscle cells in vitro. Proc Natl Acad Sci U S A 71: 1207–1210. 4208546 10.1073/pnas.71.4.1207 PMC388193

[12] Schermuly RT, Dony E, Ghofrani HA, Pullamsetti S, Savai R, et al (2005) Reversal of experimental pulmonary hypertension by PDGF inhibition. J Clin Invest 115: 2811–2821. 16200212 10.1172/JCI24838 PMC1236676

[13] Healy AM, Pickard MD, Pradhan AD, Wang Y, Chen Z, et al (2006) Platelet expression profiling and clinical validation of myeloid-related protein-14 as a novel determinant of cardiovascular events. Circulation 113: 2278–2284. 16682612 10.1161/CIRCULATIONAHA.105.607333

[14] Gnatenko DV, Cupit LD, Huang EC, Dhundale A, Perrotta PL, et al (2005) Platelets express steroidogenic 17beta-hydroxysteroid dehydrogenases. Distinct profiles predict the essential thrombocythemic phenotype. Thromb Haemost 94: 412–421. 16113833 10.1160/TH05-01-0037

[15] Lood C, Amisten S, Gullstrand B, Jonsen A, Allhorn M, et al. Platelet transcriptional profile and protein expression in patients with systemic lupus erythematosus: up-regulation of the type I interferon system is strongly associated with vascular disease. Blood 116: 1951–1957. 10.1182/blood-2010-03-274605 20538795

[16] Raghavachari N, Xu X, Harris A, Villagra J, Logun C, et al (2007) Amplified expression profiling of platelet transcriptome reveals changes in arginine metabolic pathways in patients with sickle cell disease. Circulation 115: 1551–1562. 17353439 10.1161/CIRCULATIONAHA.106.658641 PMC2225987

[17] Ambros V (2008) The evolution of our thinking about microRNAs. Nat Med 14: 1036–1040. 18841144 10.1038/nm1008-1036

[18] Bartel DP (2004) MicroRNAs: genomics, biogenesis, mechanism, and function. Cell 116: 281–297. 14744438 10.1016/s0092-8674(04)00045-5

[19] Garzon R, Pichiorri F, Palumbo T, Iuliano R, Cimmino A, et al (2006) MicroRNA fingerprints during human megakaryocytopoiesis. Proc Natl Acad Sci U S A 103: 5078–5083. 16549775 10.1073/pnas.0600587103 PMC1458797

[20] Masaki S, Ohtsuka R, Abe Y, Muta K, Umemura T (2007) Expression patterns of microRNAs 155 and 451 during normal human erythropoiesis. Biochem Biophys Res Commun 364: 509–514. 17964546 10.1016/j.bbrc.2007.10.077

[21] Bruchova H, Yoon D, Agarwal AM, Mendell J, Prchal JT (2007) Regulated expression of microRNAs in normal and polycythemia vera erythropoiesis. Exp Hematol 35: 1657–1667. 17976518 10.1016/j.exphem.2007.08.021 PMC2699372

[22] Rossi S, Shimizu M, Barbarotto E, Nicoloso MS, Dimitri F, et al. microRNA fingerprinting of CLL patients with chromosome 17p deletion identify a miR-21 score that stratifies early survival. Blood 116: 945–952. 20393129 10.1182/blood-2010-01-263889 PMC4916575

[23] Visone R, Rassenti LZ, Veronese A, Taccioli C, Costinean S, et al (2009) Karyotype-specific microRNA signature in chronic lymphocytic leukemia. Blood 114: 3872–3879. 19717645 10.1182/blood-2009-06-229211 PMC2773482

[24] Kannan M, Mohan KV, Kulkarni S, Atreya C (2009) Membrane array-based differential profiling of platelets during storage for 52 miRNAs associated with apoptosis. Transfusion 49: 1443–1450. 19389023 10.1111/j.1537-2995.2009.02140.x

[25] Landry P, Plante I, Ouellet DL, Perron MP, Rousseau G, et al (2009) Existence of a microRNA pathway in anucleate platelets. Nat Struct Mol Biol 16: 961–966. 19668211 10.1038/nsmb.1651 PMC2911476

[26] Hunter MP, Ismail N, Zhang X, Aguda BD, Lee EJ, et al (2008) Detection of microRNA expression in human peripheral blood microvesicles. PLoS One 3: e3694. 19002258 10.1371/journal.pone.0003694 PMC2577891

[27] Nagalla S, Shaw C, Kong X, Kondkar AA, Edelstein LC, et al (2011) Platelet microRNA-mRNA coexpression profiles correlate with platelet reactivity. Blood 117: 5189–5197. 21415270 10.1182/blood-2010-09-299719 PMC3109541

[28] Kondkar AA, Bray MS, Leal SM, Nagalla S, Liu DJ, et al (2010) VAMP8/endobrevin is overexpressed in hyperreactive human platelets: suggested role for platelet microRNA. J Thromb Haemost 8: 369–378. 19943878 10.1111/j.1538-7836.2009.03700.x PMC3312605

[29] Osman A, Falker K (2011) Characterization of human platelet microRNA by quantitative PCR coupled with an annotation network for predicted target genes. Platelets 22: 433–441. 21438667 10.3109/09537104.2011.560305

[30] Milosevic J, Pandit K, Magister M, Rabinovich E, Ellwanger DC, et al.. (2012) Profibrotic Role of miR-154 in Pulmonary Fibrosis. Am J Respir Cell Mol Biol. 10.1165/rcmb.2011-0377OC PMC3547095 23043088

[31] Konishi K, Gibson KF, Lindell KO, Richards TJ, Zhang Y, et al (2009) Gene expression profiles of acute exacerbations of idiopathic pulmonary fibrosis. Am J Respir Crit Care Med 180: 167–175. 19363140 10.1164/rccm.200810-1596OC PMC2714820

[32] Saldanha AJ (2004) Java Treeview–extensible visualization of microarray data. Bioinformatics 20: 3246–3248. 15180930 10.1093/bioinformatics/bth349

[33] Friedman RC, Farh KK, Burge CB, Bartel DP (2009) Most mammalian mRNAs are conserved targets of microRNAs. Genome Res 19: 92–105. 18955434 10.1101/gr.082701.108 PMC2612969

[34] Betel D, Wilson M, Gabow A, Marks DS, Sander C (2008) The microRNA.org resource: targets and expression. Nucleic Acids Res 36: D149–153. 18158296 10.1093/nar/gkm995 PMC2238905

[35] Huang da W, Sherman BT, Lempicki RA (2009) Systematic and integrative analysis of large gene lists using DAVID bioinformatics resources. Nat Protoc 4: 44–57. 19131956 10.1038/nprot.2008.211

[36] Hosack DA, Dennis G Jr, Sherman BT, Lane HC, Lempicki RA (2003) Identifying biological themes within lists of genes with EASE. Genome Biol 4: R70. 14519205 10.1186/gb-2003-4-10-r70 PMC328459

[37] Coronnello C HR, Arora A, Huleihel L, Pandit KV, et al.. (2012) Novel modeling of combinatorial miRNA targeting identifies SNP with potential role in bone density.. PLoS Comput Biol accepted. 10.1371/journal.pcbi.1002830 PMC3527281 23284279

[38] Betel D, Koppal A, Agius P, Sander C, Leslie C (2010) Comprehensive modeling of microRNA targets predicts functional non-conserved and non-canonical sites. Genome Biol 11: R90. 20799968 10.1186/gb-2010-11-8-r90 PMC2945792

[39] Kertesz M, Iovino N, Unnerstall U, Gaul U, Segal E (2007) The role of site accessibility in microRNA target recognition. Nat Genet 39: 1278–1284. 17893677 10.1038/ng2135

[40] Amisten S (2012) A rapid and efficient platelet purification protocol for platelet gene expression studies. Methods Mol Biol 788: 155–172. 22130707 10.1007/978-1-61779-307-3_12

[41] Schedel A, Rolf N (2009) Genome-wide platelet RNA profiling in clinical samples. Methods Mol Biol 496: 273–283. 18839116 10.1007/978-1-59745-553-4_17

[42] Kaushansky K (2005) The molecular mechanisms that control thrombopoiesis. J Clin Invest 115: 3339–3347. 16322778 10.1172/JCI26674 PMC1297257

[43] Queen LR, Ferro A (2006) Beta-adrenergic receptors and nitric oxide generation in the cardiovascular system. Cell Mol Life Sci 63: 1070–1083. 16568246 10.1007/s00018-005-5451-2 PMC11136379

[44] Smolenski A (2012) Novel roles of cAMP/cGMP-dependent signaling in platelets. J Thromb Haemost 10: 167–176. 22136590 10.1111/j.1538-7836.2011.04576.x

[45] Hers I (2007) Insulin-like growth factor-1 potentiates platelet activation via the IRS/PI3Kalpha pathway. Blood 110: 4243–4252. 17827393 10.1182/blood-2006-10-050633

[46] Kim S, Garcia A, Jackson SP, Kunapuli SP (2007) Insulin-like growth factor-1 regulates platelet activation through PI3-Kalpha isoform. Blood 110: 4206–4213. 17827385 10.1182/blood-2007-03-080804 PMC2234779

[47] Jison ML, Munson PJ, Barb JJ, Suffredini AF, Talwar S, et al (2004) Blood mononuclear cell gene expression profiles characterize the oxidant, hemolytic, and inflammatory stress of sickle cell disease. Blood 104: 270–280. 15031206 10.1182/blood-2003-08-2760 PMC5560446

[48] Abdollahi A (2007) LOT1 (ZAC1/PLAGL1) and its family members: mechanisms and functions. J Cell Physiol 210: 16–25. 17063461 10.1002/jcp.20835

[49] Ammons MC, Siemsen DW, Nelson-Overton LK, Quinn MT, Gauss KA (2007) Binding of pleomorphic adenoma gene-like 2 to the tumor necrosis factor (TNF)-alpha-responsive region of the NCF2 promoter regulates p67(phox) expression and NADPH oxidase activity. J Biol Chem 282: 17941–17952. 17462995 10.1074/jbc.M610618200

[50] Mendjan S, Taipale M, Kind J, Holz H, Gebhardt P, et al (2006) Nuclear pore components are involved in the transcriptional regulation of dosage compensation in Drosophila. Mol Cell 21: 811–823. 16543150 10.1016/j.molcel.2006.02.007

[51] Badeaux AI, Yang Y, Cardenas K, Vemulapalli V, Chen K, et al (2012) Loss of the methyl lysine effector protein PHF20 impacts the expression of genes regulated by the lysine acetyltransferase MOF. J Biol Chem 287: 429–437. 22072714 10.1074/jbc.M111.271163 PMC3249094

[52] Benetatos L, Hatzimichael E, Londin E, Vartholomatos G, Loher P, et al.. (2012) The microRNAs within the DLK1-DIO3 genomic region: involvement in disease pathogenesis. Cell Mol Life Sci. 10.1007/s00018-012-1080-8 PMC11114045 22825660

[53] Seitz H, Royo H, Bortolin ML, Lin SP, Ferguson-Smith AC, et al (2004) A large imprinted microRNA gene cluster at the mouse Dlk1-Gtl2 domain. Genome Res 14: 1741–1748. 15310658 10.1101/gr.2743304 PMC515320

[54] Dixon-McIver A, East P, Mein CA, Cazier JB, Molloy G, et al (2008) Distinctive patterns of microRNA expression associated with karyotype in acute myeloid leukaemia. PLoS One 3: e2141. 18478077 10.1371/journal.pone.0002141 PMC2373886

[55] Steele BM, Harper MT, Macaulay IC, Morrell CN, Perez-Tamayo A, et al (2009) Canonical Wnt signaling negatively regulates platelet function. Proc Natl Acad Sci U S A 106: 19836–19841. 19901330 10.1073/pnas.0906268106 PMC2785253

[56] Toma I, McCaffrey TA (2012) Transforming growth factor-beta and atherosclerosis: interwoven atherogenic and atheroprotective aspects. Cell Tissue Res 347: 155–175. 21626289 10.1007/s00441-011-1189-3 PMC4915479

[57] Bazzoni G (2011) Pathobiology of junctional adhesion molecules. Antioxid Redox Signal 15: 1221–1234. 21254840 10.1089/ars.2010.3867

[58] Nachman RL, Rafii S (2008) Platelets, petechiae, and preservation of the vascular wall. N Engl J Med 359: 1261–1270. 18799560 10.1056/NEJMra0800887 PMC2935201

[59] Chavda N, Mackie IJ, Porter JB, Harrison P, Patterson K, et al (1996) Rapid flow cytometric quantitation of reticulated platelets in whole blood. Platelets 7: 189–194. 21043687 10.3109/09537109609023578

[60] Freedman ML, Karpatkin S (1975) Elevated platelet count and megathrombocyte number in sickle cell anemia. Blood 46: 579–582. 1174691

[61] Noronha JF, Costa FF, Saad ST, Lorand-Metze IG, Grotto HZ (2007) Evaluation of reticulated platelets in patients with sickle cell diseases. Thromb Res 121: 259–267. 17521711 10.1016/j.thromres.2007.04.002

[62] Popescu ER, Martelo OJ (1977) Megathrombocytes and sickle cell anemia. Blood 49: 490–491. 836958

[63] Berckmans RJ, Nieuwland R, Boing AN, Romijn FP, Hack CE, et al (2001) Cell-derived microparticles circulate in healthy humans and support low grade thrombin generation. Thromb Haemost 85: 639–646. 11341498

[64] Horstman LL, Ahn YS (1999) Platelet microparticles: a wide-angle perspective. Crit Rev Oncol Hematol 30: 111–142. 10439058 10.1016/s1040-8428(98)00044-4

[65] Joop K, Berckmans RJ, Nieuwland R, Berkhout J, Romijn FP, et al (2001) Microparticles from patients with multiple organ dysfunction syndrome and sepsis support coagulation through multiple mechanisms. Thromb Haemost 85: 810–820. 11372673

