# Supplementary figures and images for: Expression of Regulatory Platelet MicroRNAs in Patients with Sickle Cell Disease
Source: PLoS One. 2013 Apr 12;8(4):e60932. doi: 10.1371/journal.pone.0060932 (PMC3625199; doi:10.1371/journal.pone.0060932)

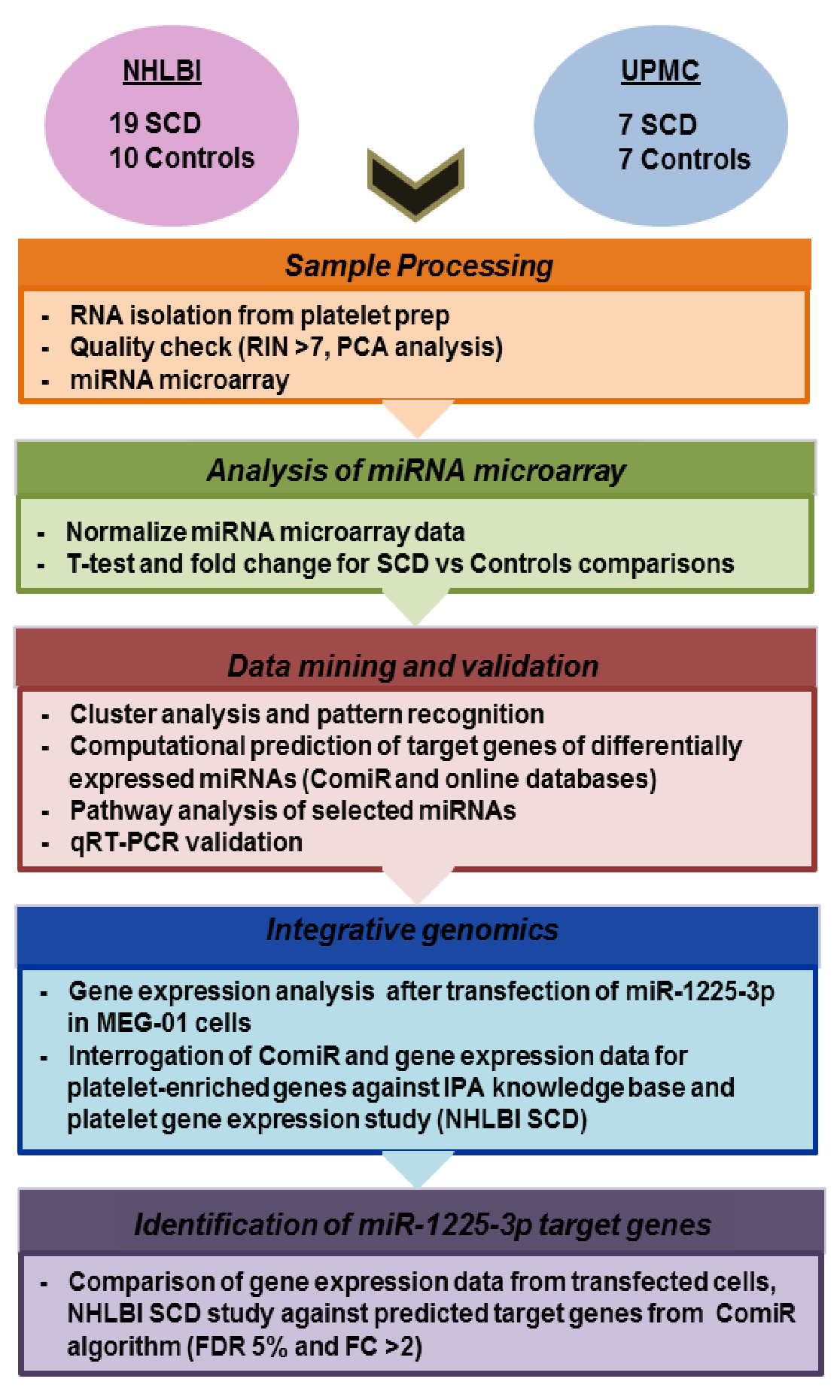

Supplement: Figure S1 — Workflow for the bioinformatics analyses used in the study for predicting target genes of miR-1225-3p. (TIF) [file pone.0060932.s001.tif]

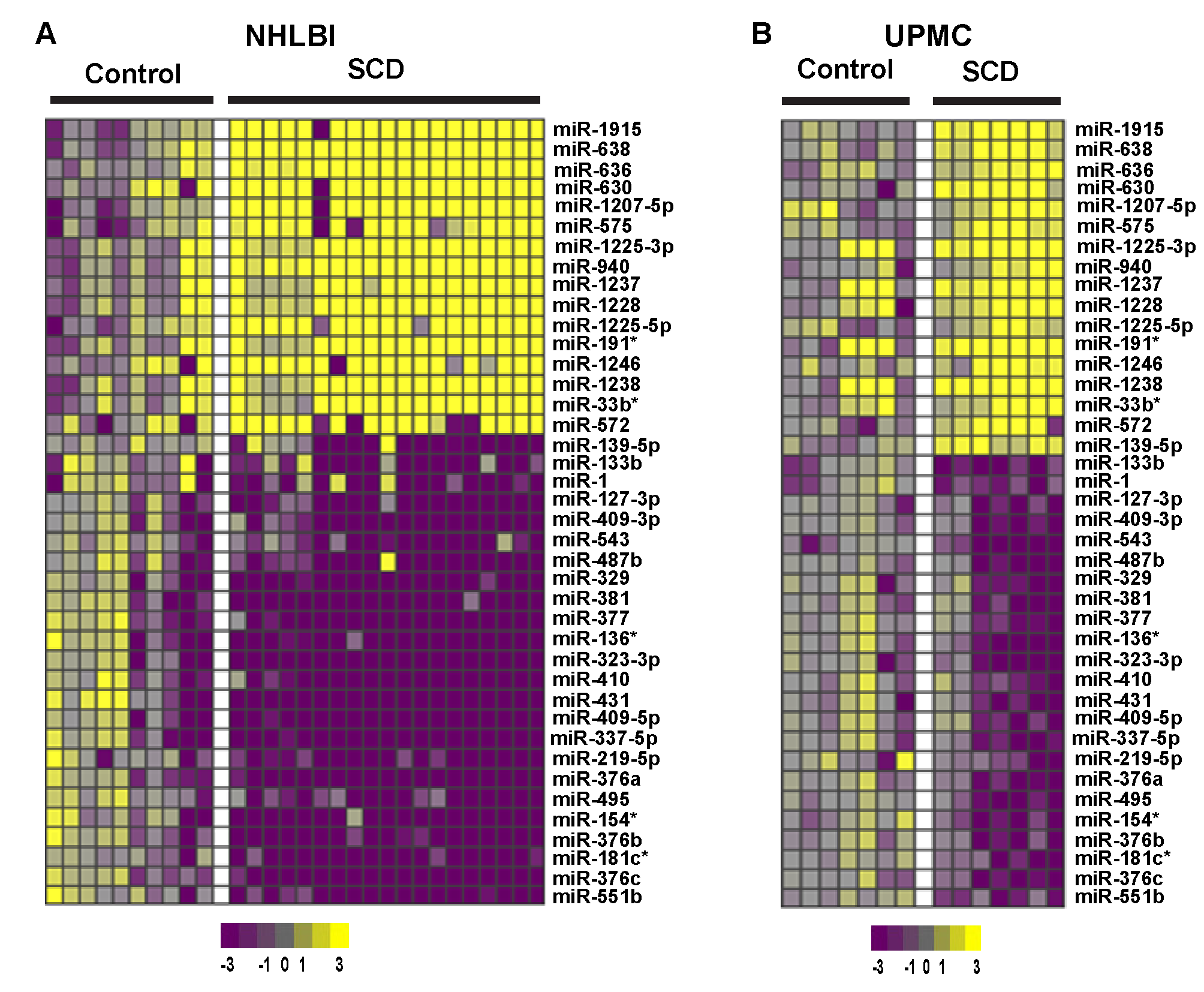

Supplement: Figure S2 — Two heatmaps for each of the cohorts-UPMC and NHLBI, depicting the 40 statistically significant (p-value<0.05, FC>2) differentially expressed miRNAs between SCD and controls. The two cohorts display consistency in the directionality of the differentially expressed miRNAs between controls and SCD samples. Columns represent individual samples and each represents a miRNA. Upregulated miRNAs expression levels are shown in progressively brighter shades of yellow, depending on the fold difference. Downregulated miRNAs are shown in progressively brighter shades of purple. No difference is represented as grey. The names of the miRNAs are displayed to the right of the heatmap. (TIF) [file pone.0060932.s002.tif]

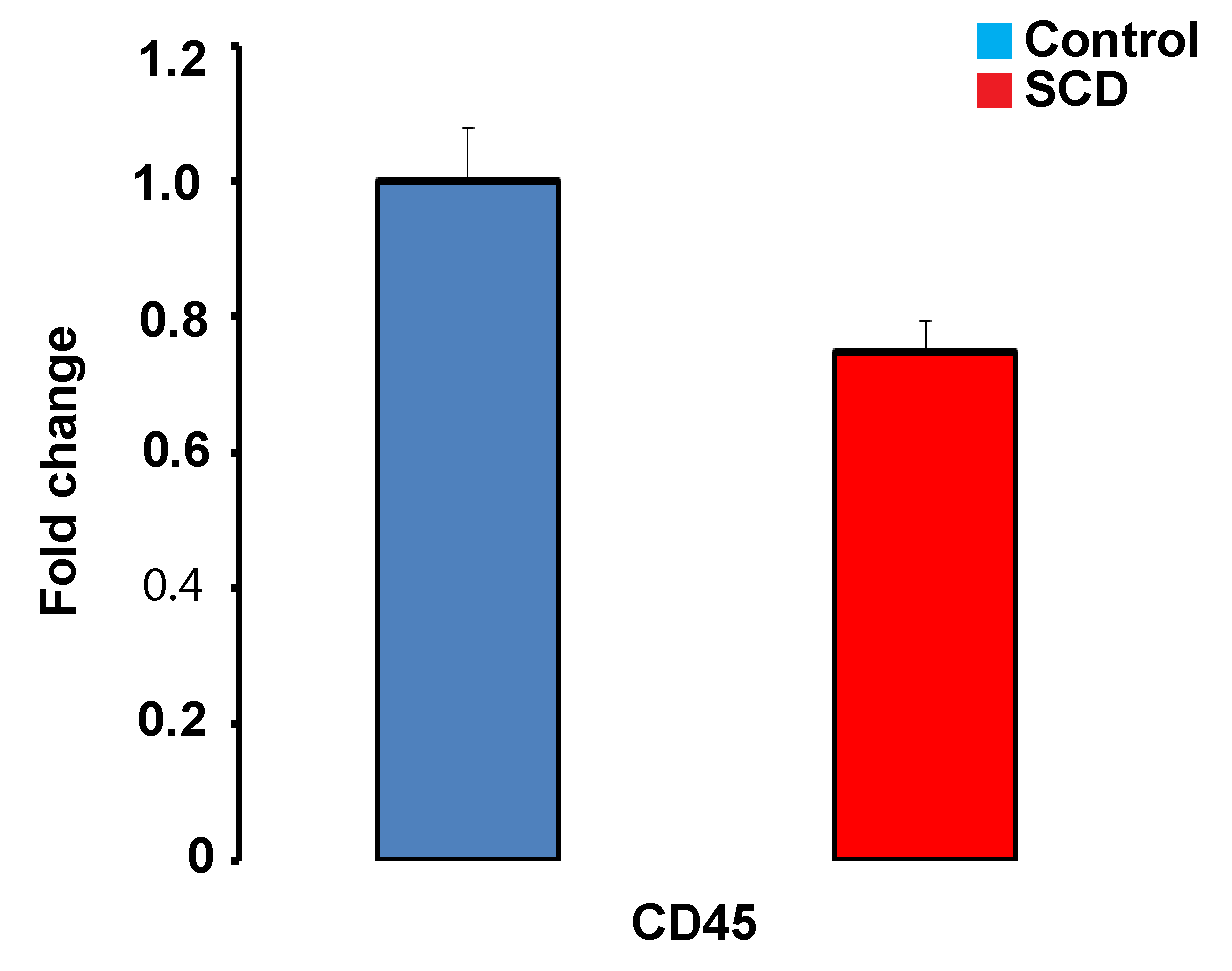

Supplement: Figure S3 — Expression of leukocyte-specific CD45/PTPRC by RT-PCR in SCD and control platelet samples did not reveal a significant difference. For each miRNA the blue bar represents controls and the red bar represents SCD. Y-axis shows fold change of miRNAs in SCD samples with the expression level in controls set to 1. Error Bars represent standard deviation. (TIF) [file pone.0060932.s003.tif]
