# Supplementary material for: Expression of Regulatory Platelet MicroRNAs in Patients with Sickle Cell Disease
Source: PLoS One. 2013 Apr 12;8(4):e60932. doi: 10.1371/journal.pone.0060932 (PMC3625199; doi:10.1371/journal.pone.0060932)
Supplement: Table S2 — List of differentially expressed genes (FDR<5%) in transfected cells vs scrambled cells with functional annotations pertaining to platelet and megakaryocytes (n = 75) based on IPA knowledge base. (DOCX) [file pone.0060932.s005.docx]

| **Function Annotation** | **Gene symbol** |
| --- | --- |
| **Affects, Increases or Decreases**  **differentiation of megakaryocytes** | BAIAP2, E2F1, FI1B, FLI1, GATA2, HMGB2, JAK2, NFE2, OSM, RCOR1, RPS6KA1, TESC, WASF2 |
| **Increases quantity of megakaryocyte / erythrocyte**  **lineage-restricted progenitor cells** | FANCA |
| **Increases quantity of promegakaryocytes** | INPP5D |
| **Affects abnormal morphology of megakaryocyte / erythrocyte lineage-restricted progenitor cells** | FLI1, SENP1, TP53 |
| **Affects, Increases or Decreases quantity / production / formation of blood platelets** | ADD1, ARNTL, ANXA7, BAK1, EGR1, FCGR2A, FLI1, IL6, IL11, IL1A, IL1B, INPP5D, LYN, MDM4, MLL5, NFE2, PROS1, SH2B3, STIM1, THBS1, TP53, TSPAN33, VPS33A, WASF2, ZC3H12A |
| **Affects, Increases or Decreases**  **aggregation of blood platelets** | ADORA2B, AKT3, ANXA7, BTK, CAST, CD151, CLNS1A, F2R, FCER1G, GSK3B, GUCY1A3, INPP5D, ITGAV, LYN, MET, PIK3CG, PLCB2, PLCB3, PTPN1, PTPN9, THBS1, TIMP2, VAV1 |
| **Affects, Increases or Decreases**  **adhesion of blood platelets** | FCER1G, PPIB, THBS1,VASP |
| **Affects, Increases or Decreases**  **activation of blood platelets** | CD24, CD59, CTSG, F2R, FCER1G, INPP5D, IL6, LYN, PPAP2A, PRG2, PTPRJ, SELPLG, STIM1, THBS1 |
| **Increases or Decreases cell spreading**  **Increases cell movement and rolling of**  **blood platelets** | CAST, FCGR2A, LYN, SELPLG, THBS1 |
| **Affects function of blood platelets** | BAK1, BTK, CD59, F2R, PLCB3, PLCB2, PTGS1, PTPN9 |
| **Increases binding of blood platelets** | CD24, CD84, NFE2, SELPLG |
| **Affects, Increases or Decreases shape change or morphology of blood platelets** | BAK1, F2R, CAST, FCGR2A, LYN, NFE2, THBS1 |
| **Affects cell-cell contact of blood platelets** | INPP5D |
| **Affects recruitment of blood platelets** | VASP |
| **Increases/affects engulfment or phagocytosis of blood platelets** | THBS1 |
| **Increases or Decreases synthesis of platelet**  **activating factor** | ALOX5, FCGR2A, IL1B, PAFAH1B1  SLC6A6 |
| **Affects, Increases or Decreases**  **Thrombocytopathy** | APP, BAK1, E2F1, EGR1, F2R, FCER1G, FLI1, IFNGR1, INPP5D, STIM1 |

**Table S2. List of differentially expressed genes (FDR <5%) in transfected cells vs scrambled cells with functional annotations pertaining to platelet and megakaryocytes (n=75) based on IPA knowledge base.**
